# A squalene analog 4,4′-diapophytofluene from coconut leaves having antioxidant and anti-senescence potentialities toward human fibroblasts and keratinocytes

**DOI:** 10.1038/s41598-024-63547-1

**Published:** 2024-06-01

**Authors:** Madhurima Dutta, Swarupa Sarkar, Parimal Karmakar, Suparna Mandal Biswas

**Affiliations:** 1 Agricultural and Ecological Research Unit, Indian Statistical Institute, 203, B.T. Road, Kolkata, 700108 India; 2 Department of Life Science and Biotechnology, Jadavpur University, 188, Raja Subodh Chandra Mallick Rd, Kolkata, 700032 India

**Keywords:** Squalene, 4,4′-Diapophytofluene (4,4′-DPE), Cellular senescence, Cytotoxicity, Reactive oxygen species (ROS), Senotherapeutic agent, Biochemistry, Biotechnology, Cell biology, Chemical biology, Drug discovery, Molecular biology, Health care

## Abstract

Coconut (*Cocos nucifera)* leaves, an unutilized resource, enriched with valuable bioactive compounds. Spectral analysis of purified pentane fraction of coconut leaves revealed the presence of a squalene analog named 4,4′-diapophytofluene or in short 4,4′-DPE (C_30_H_46_). Pure squalene standard (PSQ) showed cytotoxicity after 8 µg/ml concentration whereas 4,4′-DPE exhibited no cytotoxic effects up to 16 µg/ml concentration. On senescence-induced WI38 cells, 4,4′-DPE displayed better percentage of cell viability (164.5% at 24 h, 159.4% at 48 h and 148% at 72 h) compared to PSQ and BSQ (bio-source squalene) with same time duration. Similar trend of result was found in HaCaT cells. SA-β-gal assay showed that number of β-galactosidase positive cells were significantly decreased in senescent cells (WI38 and HaCaT) after treated with 4,4′-DPE than PSQ, BSQ. Percentage of ROS was increased to 60% in WI38 cells after olaparib treatment. When PSQ, BSQ and 4,4′-DPE were applied separately on these oxidative-stress-induced cells for 48 h, the overall percentage of ROS was decreased to 39.3%, 45.6% and 19.3% respectively. This 4,4′-DPE was found to be more effective in inhibiting senescence by removing ROS as compared to squalene. Therefore, this 4,4′-DPE would be new potent senotherapeutic agent for pharmaceuticals and dermatological products.

## Introduction

Ageing^1^ is a progressive process of decline in the physiological functions of tissues and organs, leading toward the debility of overall fitness of an organism. At the cellular level, cells show permanent growth arrest or replicative senescence and reduced autophagy with aging. Most of these senescent cells (SnCs) develop unique biological features called senescent-associated secretory phenotypes (SASPs). These SASPs are able to stimulate chronic inflammation and tissue dysfunction by secreting pro-inflammatory compounds, chemokines, cytokines, proteases, bioactive lipids, extracellular vesicles, metabolites, inhibitory molecules and many other factors^2^. Therefore, the reduction or killing of SnCs and enhanced autophagy are directly related with rejuvenation and healthy aging. Natural products can influence the cellular activity toward senescence and autophagy^3^. However, there are only a limited number of compounds that can function as senotherapeutics, either by killing these SnCs, or modulate them morphologically and functionally into normal cells, or delaying this senescence event^4^. Therefore, the applications of these senotherapeutics can be the modern-day approach to healthy aging.

Ageing can be visibly observed on human skin as fine lines and wrinkles due to skin oxidation and accumulation of SnCs. The imbalance between the generation of reactive oxygen species (ROS) and their neutralization, accelerated the ageing process^5^. Several studies indicated that the free radical neutralizing ability of plant-derived polyphenols, tocopherols, carotenoids etc. and other essential oil are proficient to maintain good skin health^6^. Among the natural products, squalene and its derivatives were selected for this present study based on their unique characteristics.

Squalene (SQ) is an important polyunsaturated triterpene, which is synthesized through mevalonate pathway or methylerythritol pathway by squalene synthase (SQS)^7^. Although this squalene synthase (SQS) can directly produce dehydrosqualene as a byproduct in the absence of NADPH or due to the activity of dehydrosqualene synthase (crtM)^8^. Dehydrosqualene further enters into the C_30_ carotenoid biosynthesis by producing carotenoid pigments such as 4,4′-diapophytoene, 4,4′-diapophytofluene (4,4′-DPE), 4,4′-diapo-ζ-carotene and 4,4′-diaponeurosporene, respectively^9^. These yellow to orange pigments are known to decrease the risk for cardiovascular diseases, many types of cancer, UV-induced photosensitivity and also age-related macular degeneration. They also possess imperative biological features related to their antioxidant activity, provitamin-A activity, ability to regulate gene transcription and enhancement of gap junction communication^10^.

Squalene (SQ) acts as an antioxidant that are mostly synthesized in the sebaceous gland of the human skin and its content is significantly decreased with ageing^11^. SQ can form a hydration barrier in a comparable manner to vernix caseosa and thus its use as a natural emollient for skin has been increased immensely in cosmetic products^12^. However, there is no experimental evidence on squalene and its derivatives activity addressing the deceleration ageing or age-related ailment in human cells. Especially its role in preventing cellular senescence, a predominant phenomenon during cellular ageing, has not been studied so far.

The main source of squalene was deep sea shark liver oil (40–70%), which was leading to over exploitation of shark species by cruel hunting process^13^. Stringent regulations have been employed by International Fishing Authorities and Commission for Conservation of Antarctic Marine Living Resources towards shark livering and finning. These lead the researchers to find alternative vegan sources of SQ. There are some reported plant sources of SQ such as olive oil, palm oil, rice bran oil, amaranthus seed oil, soybean etc.^14^. However, these vegan sources of squalene contribute a very little amount to fulfill the present global demand.

*Bactris gasipaes* Kunth. (Peach Palm) from Arecaceae family was found to possess 17% squalene of total unsaponifiable seed oil^15^. Another two palm plants from this same family namely *Arenga tremula* and *Arenga engleri*, have also been reported to contain squalene in their leaves ^16,17^. Based on this fact, coconut (*Cocos nucifera* L.) leaves were chosen for this study as a tested resource as it is a very common plant of the family Arecaceae and ubiquitously occurred all over India^18^. Coconut leaves are generally used as roofing material or for making brooms in rural areas. Besides this, they have no such commercial applications. Therefore, this can be a sustainable source for extracting biological compounds.

The aim of this present research was to evaluate the role of vegan squalene and its derivatives in the reduction or killing of senescent cells for rejuvenation and healthy aging. Therefore, cytotoxic activity, anti-senescence activity and ROS scavenging activity of squalene and its derivatives were performed following standard protocols.

## Materials and methods

### Collection of plant samples

Leaves of *Cocos nucifera,* commonly known as coconut plant, were collected from the nearby areas of Kolkata (22.6482° N, 88.3768° E), West Bengal, India, from October to December in the year 2021. Plant samples were collected in accordance with plant guidelines for plant sampling. Fresh non-infected leaves were selected and surface sterilized. After that, stiff central midribs were removed from *C. nucifera* leaves and the remaining portions were dried in air at room temperature (25 ± 2 °C) under the shed for 3–4 days. Leaves of *Artocarpus lakoocha* were collected from the nearby areas of Kolkata, for the extraction of bio-source squalene (BSQ)^19^.

### Isolation and purification of squalene and its derivates

About 100 gm of air-dried leaves of *C. nucifera* were taken and minced in Sample Miller Machine and then soaked in 500 ml n-pentane in a 2000 ml of extraction flask for 2–3 days. The extract was collected and treated with activated silica gel (60–120 mesh) by vigorous shaking for removing the impurities. The impurities were stored in the lower silica gel and upper filtrate containing squalene or its derivates. The upper filtrate was collected in another fresh flask and treated again with silica gel. This procedure was repeated 3 to 4 times until the filtrate became colorless. Bio-source squalene (BSQ) extraction was carried out from leaves of *A. lakoocha* following the same procedure. The concentrated crude sample was purified further by running it through the silica column using pentane as the mobile phase. For getting the primary idea, *C. nucifera* pentane extract and pure squalene standard (Sigma-Aldrich, ≥ 98%) were then applied on a thin layer chromatography (TLC Silica gel 60 F_254_) plate and run in pentane solvent only and spots were visualized under iodine vapour.

### General experimental procedures

#### LC-HRMS analysis

Purified pentane fraction of *C. nucifera* leaves were subjected to LC-HRMS (Liquid chromatography-high resolution mass spectrometry)^20^ analysis had been done with Agilent Technologies, USA, Model: 6550 iFunnel Q-TOFs for detecting metabolomics profiling at the Sophisticated Analytical Instrument Facility, Indian Institute of Technology (IIT), Bombay, India. The identification of compounds was executed by comparing the mass spectra with the spectral data of the NBS75K library (The NIST Mass Spectrometry Data Center) provided by the LC-HRMS data processing software. ^13^*C- and *^*1*^*H-NMR analysis:* ^13^C and ^1^H NMR (600 MHz) analyses of highly purified pentane fraction of *C. nucifera* was recorded with a JEOL, Japan, Model: JNM-ECZ600R/S1 spectrometer available at Sophisticated Analytical Instrument Facility, IIT, Bombay, India. ^13^C NMR spectra was recorded on δ ppm (0–200) scale with an end sweep at 0 ppm; whereas, ^1^H NMR spectra were noted on δ ppm (0–10) scale with end sweep at 0 ppm by using single pulse program. This sample was analyzed at ambient temperature, using chloroform (CDCl_3_) as solvent. Fourier Transform Infrared Spectroscopy (FTIR) analysis*:* FTIR analysis (at ATR mode) of highly purified pentane fraction of *C. nucifera* was performed by FTIR Spectrophotometer (Model: Tensor 27 Bruker, Germany) available at the IICB, Kolkata, India for detecting the types of functional groups present in the compound.

### MTT assay for measuring cytotoxicity

Normal WI38 cells were seeded in 48 well plates for 20 h. After that these cells were treated with pure squalene standard or PSQ (Sigma-Aldrich), bio-source squalene (BSQ) and the compound extracted from *C. nucifera* leaves (4,4′-DPE) at different concentrations (0.1% DMSO) for 24 h. Next day, 300 µl of MTT (3-[4,5-dimethylthiazol-2-yl]-2,5 diphenyl tetrazolium bromide) solution was added and incubated for 4 h. Then carefully remove the medium without disturbing the crystals and washing was done by using phosphate buffer saline (PBS). Finally, the resulting formazan crystals were dissolved and the plate was read by using a microplate reader (Epoch Micro-plate Spectrophotometer, USA) at 570 nm^21^. Untreated cells were considered as 100% viable.

### Cellular viability assessment of senescence-induced cells

The growth of senescence-induced cells was estimated by following MTT assay as described earlier. This time, two different normal WI38 and HaCaT cells were seeded separately in 48 well plates for 20 h. Senescence was induced in the mentioned cell lines by using olaparib (5 µg/ml). Then they were treated with PSQ, BSQ and 4, 4'-DPE of 8 µg/ml (0.1% DMSO) for 24–72 h. MTT solution was added and cell viability was assessed as described above.

### Senescence Associated-β-galactosidase (SA-β-gal) assay

SA-β-gal assay^22^ was done to evaluate their activity in the prevention of cellular senescence, which is directly related with the organismal aging. WI38 and HaCaT cells were seeded separately in coverslips for 20 h. Senescence was induced in the mentioned cell lines by using olaparib (5 µg/ml). Then they were treated with PSQ, BSQ and 4,4′-DPE extract of 8 µg/ml for 24 h. The next day, cells were fixed by using 4% paraformaldehyde followed by ß-galactose staining using the method described elsewhere.

### Intracellular ROS scavenging assay

Intracellular reactive oxygen species (ROS) scavenging activity of PSQ, BSQ and 4,4′-DPE in WI38 cells was detected by ROS assay^23^, another important marker of cellular senescence. For this assay, a non-fluorescent probe named 2',7'-dichlorodihydrofluorescein diacetate (H2DCFDA: Thermo-Fisher scientific) has been used. The cells of WI38 were cultured in petri dishes for 24 h. After that senescence was induced in the mentioned cell lines by 5 µg/ml olaparib for another 24 h. Then 100 μg/ml of 2',7'-dichlorodihydrofluorescein diacetate (DCFDA) was added to the medium for 30 min at 37˚C in the dark. In the other experimental sets, PSQ, BSQ and 4,4′-DPE were added at 8 μg/ml concentration to the olaparib treated cell lines for 48 h followed by application of 100 μg/ml DCFDA for 30 min. DCFDA diffuses through the cell membrane, enzymatically hydrolysed by intracellular esterases and oxidized to produce a green fluorescent compound called 2',7'-dichlorofluorescein (DCF) in the presence of ROS. Cells were visualized by fluorescence microscope at 529 nm after rinses with PBS. The intensity of fluorescence is directly related to the level of intracellular ROS.

### Statistical analysis

All biological experiments were performed in triplicates and values were represented in mean with standard deviation. One-way analysis of variance (ANOVA) followed by Tukey-HSD multiple comparison test was undertaken to confirm data variability and significance of the results (p < 0.05) of treatment dependent response of the selected cell line. The Student's t-test had been done to compare the means between cell viability and time (p < 0.05). All statistical analyses were carried out using SPSS-18^24^ software package.

## Results

### Isolation and purification of biosource squalene and its derivatives

About 323 mg of the desired compound with 99% purity was recovered from 100 g of leaf dust of *Cocos nucifera* L. Thin Layer Chromatographic (TLC) run of pure squalene standard (Sigma-Aldrich), bio-source squalene extracted from *Artocarpus lakoocha* leaves^19^ (BSQ) and purified pentane extract of coconut leaves, exhibited spots with same R_f_ value (6.8) under iodine vapour. So, it indicated that the compound present in these extracts would be a more or less similar category.

### Identification of 4,4′-diapophytofluene through spectral analysis

**Figure 1:**
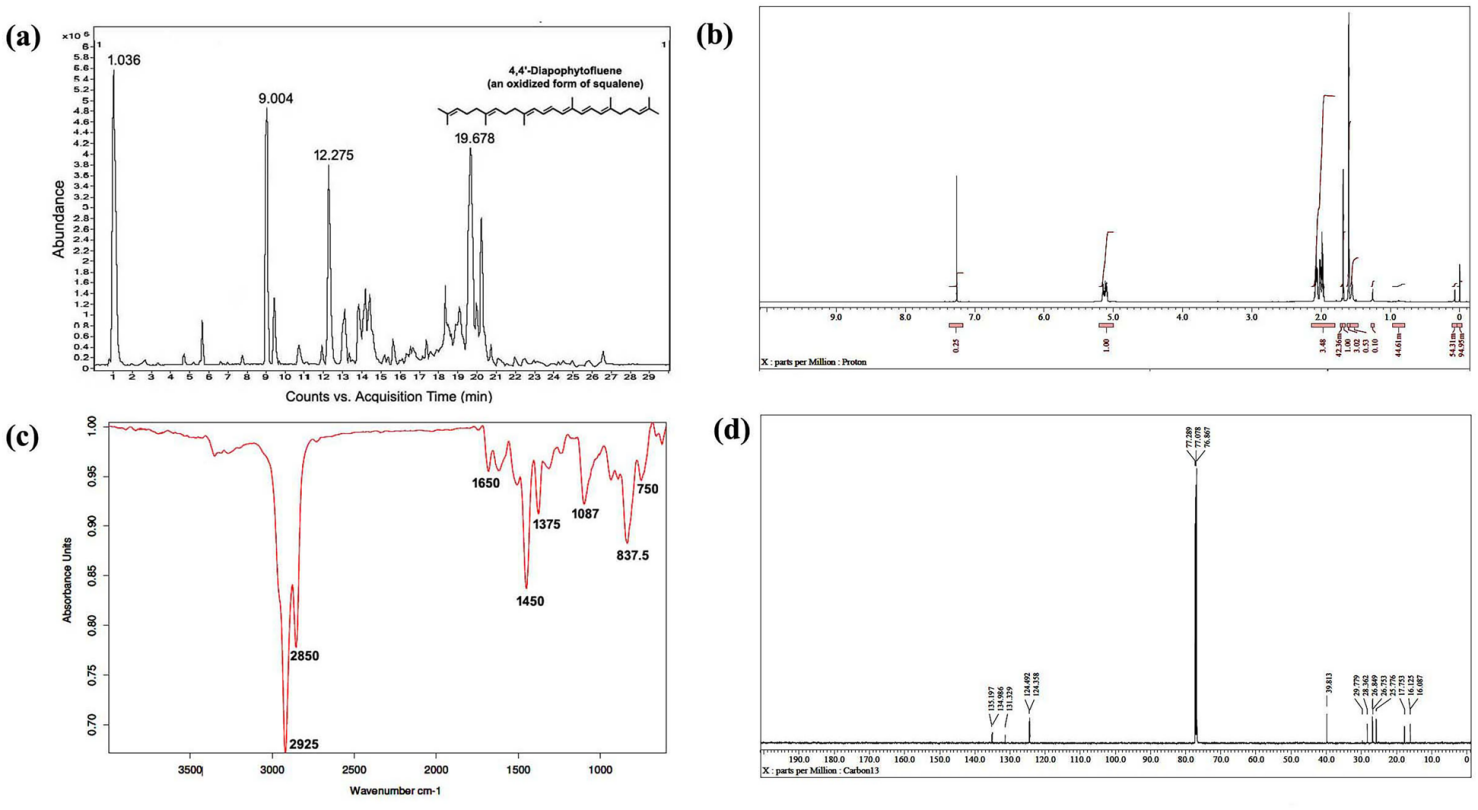
Spectral analysis of purified pentane fraction of dried *Cocos nucifera* L. leaves. (**a**) Mass spectra, (**b**) ^1^HNMR spectra, (**c**) FTIR spectra, (**d**) ^13^CNMR spectra.

**Figure 2:**
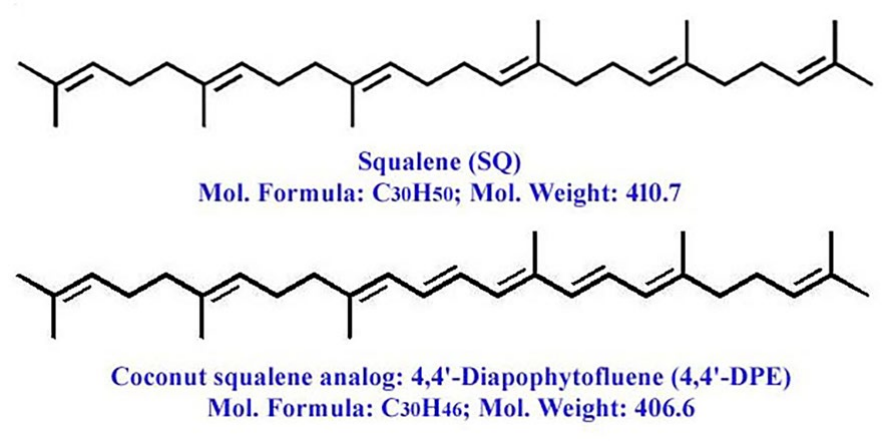
Molecular structure of pure squalene standard (PSQ) and squalene analog from *Cocos nucifera* L. or 4,4′-diapophytofluene (4,4′-DPE).

^13^CNMR spectra revealed the presence of 8 methyl groups, 10 methylene groups, 8 double bonds and the rest are for CH carbons. The compound is indeed in dimeric form. The ^13^C peaks at δ 135.197, 134.986 and 131.329 ppm showed the existence of C carbon. Peaks at δ 124.492 to 124.358 ppm are for CH carbon. The ^13^C peaks at δ 39.813, 29.779, 28.362, 26.849 and 26.753 ppm supported the presence of 10 methylene carbon. Peaks at δ 25.776, 17.753, 16.125 and 16.087 ppm suggested the existence of 8 methyl carbon (Fig. 1d).

LC-HRMS spectra of the desired compound revealed four major peaks with retention time 1.036, 9.004, 12.275 and 19.678. Of which the compound named 4,4′-diapophytofluene or 4, 4'-DPE (R_t_ = 19.678 min) with molecular formula C_30_H_46_ showed much similarity with the squalene structure (Fig. 1a). The molecular weight of the desired compound (4,4′-DPE) is 406 whereas the molecular weight of pure standard squalene (PSQ) is 410. Both have the same chemical structure except that the compound found in the coconut leaves extract has two extra double bonds at the 8th and 12th carbon position of their molecular structure (Fig. 2).

In ^1^HNMR spectra, peak at δ 1.72 ppm suggested the presence of methyl hydrogen and δ 1.95 to δ 2.12 revealed the occurrence of methylene hydrogen (Fig. 1b). Peak from δ 5.16 to δ 5.08 ppm indicated ethylene hydrogen (Supplementary Figs. S1 and S2).

FT-IR absorption spectra of purified of pentane fraction of coconut leaves extract revealed eight distinct peaks. Peak at 2925 cm^−1^ and 2855 cm^−1^ were attributed to methylene asymmetric (*v*_*as*_CH_2_) and symmetric (*v*_*s*_CH_2_) stretching. Peak at 1650 cm^−1^ suggested C=C stretching vibration of unconjugated linear alkene. Asymmetrical bending vibration (δ_as_) and symmetrical bending vibration (δ_s_) of methyl group appeared at 1450 cm^−1^ and 1375 cm^−1^ respectively. Carbon-hydrogen bending vibrations of the =C–H group occurred in the region 1000–650 cm^−1^ (Fig. 1c).

### Assessment of cellular viability of senescence-induced cells after application of squalene and its analog

First, MTT assay was done to detect the cytotoxic effects of these compounds on normal cells. The percentage of cell viability was increased with the increasing concentrations of 4,4′-DPE up to 16 μg/ml (151.4%) and beyond this concentration cell viability declined (Supplementary Fig. S3b). But in case of PSQ and BSQ percentage of cell viability increased up to 8 μg/ml concentration (121% and 115.4% respectively) and after that cell viability percentage rapidly decreases (Supplementary Fig. S3a). Thus, 4,4′-DPE mainly showed a better cell viability percentage (146.95%) than PSQ and BSQ and the concentration of 8 μg/ml was selected as the compound dose for the following in vitro experiments.

**Figure 3:**
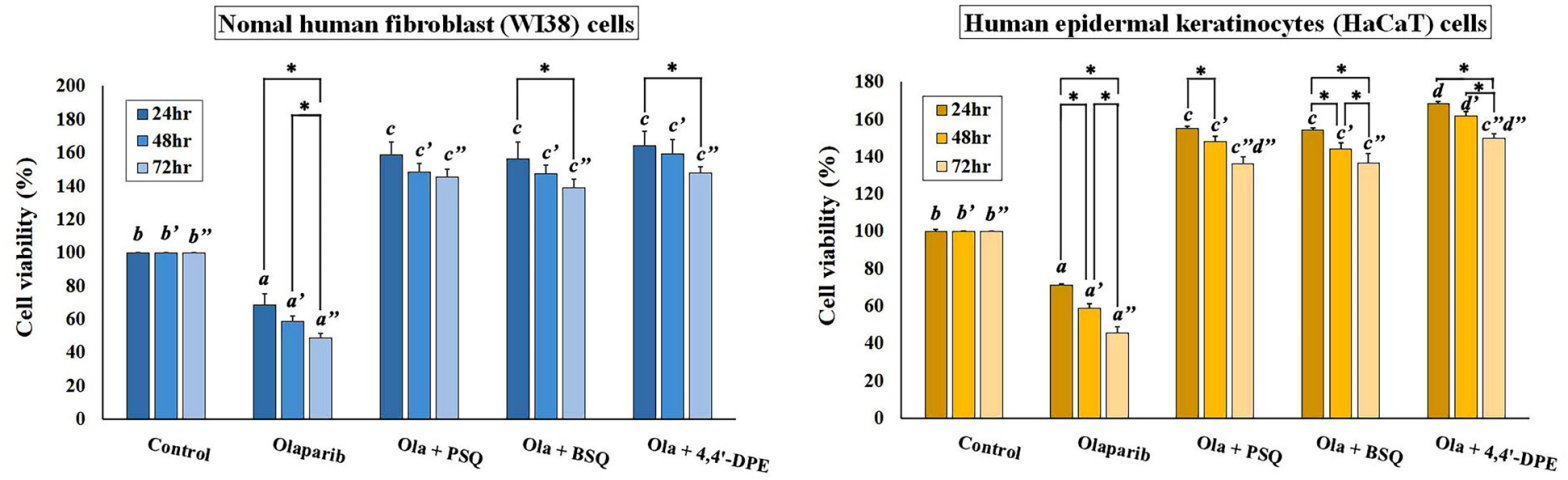
Effects of pure squalene standard (PSQ), bio-source squalene extracted from *Artocarpus lakoocha* L. leaves (BSQ) and squalene analog or 4,4′-diapophytofluene (4,4′-DPE) from *Cocos nucifera* L. at 8 µg/ml concentration on senescence induced cells (by 5 µg/ml Olaparib) of normal human fibroblast (WI38) cells and human epidermal keratinocytes (HaCaT) cells with different time duration. Data represents the mean of three replicates and error bars represent standard deviation. Asterisk indicates p < 0.05.

Another MTT assay was set up to explore the anti-senescent activity of these compounds on olaparib (a senescence inducer drug) treated WI38 and HaCaT cells at 8 μg/ml concentration. From this particular assay, it was observed that the percentage of cell viability significantly decreased to 68.8% in WI38 cells and 71.1% in HaCaT cells after 24 h of olaparib treatment (Fig. 3). However, the percentage of viable cells had been increased after the cells were treated separately with PSQ, BSQ and 4,4′-DPE. In case of WI38 cells, 4,4′-DPE exhibited a greater percentage of cell viability (i.e., 164.5% at 24 h, 159.4% at 48 h and 148% at 72 h) than PSQ (158.7% at 24 h, 148.2% at 48 h and 145.5% at 72 h) and BSQ (156.5% at 24 h, 147.6% at 48 h and 138.8% at 72 h). Whereas in HaCaT cells, 4, 4'-DPE also showed the better percentage of cell viability (168.4% at 24 h, 161.8% at 48 h and 149.9% at 72 h) than PSQ (155.3% at 24 h, 148.1% at 48 h and 136.1% at 72 h) and BSQ (154.2% at 24 h, 143.9% at 48 h and 136.6% at 72 h) with different time duration. Overall, the application of 4,4′-DPE on senescence induced HaCaT cells displayed significantly greater cellular viability percentage up to 48 h than the other two treatments.

### Inhibition of induced senescence on WI38 and HaCaT cells through SA-ß-gal assay.

**Figure 4:**
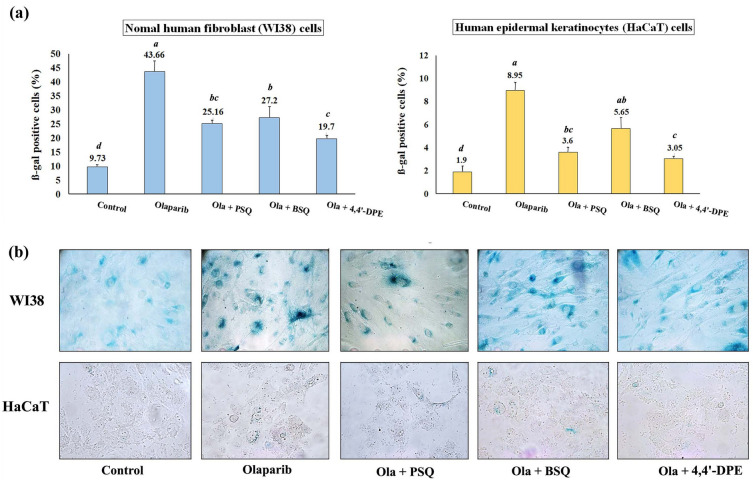
Senescence-associated beta-galactosidase assay on normal human fibroblast (WI38) and human epidermal keratinocytes (HaCaT) after exposure to 5 µg/ml olaparib for 24 h followed by application of 8 µg/ml of each test samples i.e., pure squalene standard (PSQ), bio-source squalene extracted from *Artocarpus lakoocha* L. leaves (BSQ) and squalene analog or 4,4′-diapophytofluene (4,4′-DPE) from *Cocos nucifera* L. for 48 h. (**a**) Graphical representation and (**b**) pictorial representation. Data represents the mean of three replicates and error bars represent standard deviation. Letters ‘a’, ‘b’, ‘c’, ‘d’ indicate significant differences (Turkey’s HSD multiple comparison at p < 0.05).

SA-ß-gal assay indicated that the percentage of ß-gal positive cells were significantly increased to 43.7% and 8.95% respectively for WI38 and HaCaT cells after olaparib treatment. When these senescence induced cells were treated independently with PSQ, BSQ and 4,4′-DPE, the amount of ß-gal positive cells had been reduced to 25.2%, 27.2% and 19.7% respectively in WI38 cells. Likewise, in HaCaT cells, the percentage of ß-gal positive cells was lowered to 3.6%, 5.65% and 3.05% respectively (Fig. 4). From this result, it is evident that treatment of 4,4′-DPE showed significantly better senescence inhibiting activity than BSQ. However, 4,4′-DPE exhibited almost similar activity like that of PSQ.

### Intracellular ROS scavenging activity of squalene and its analog on senescence-induced WI38 cells

**Figure 5:**
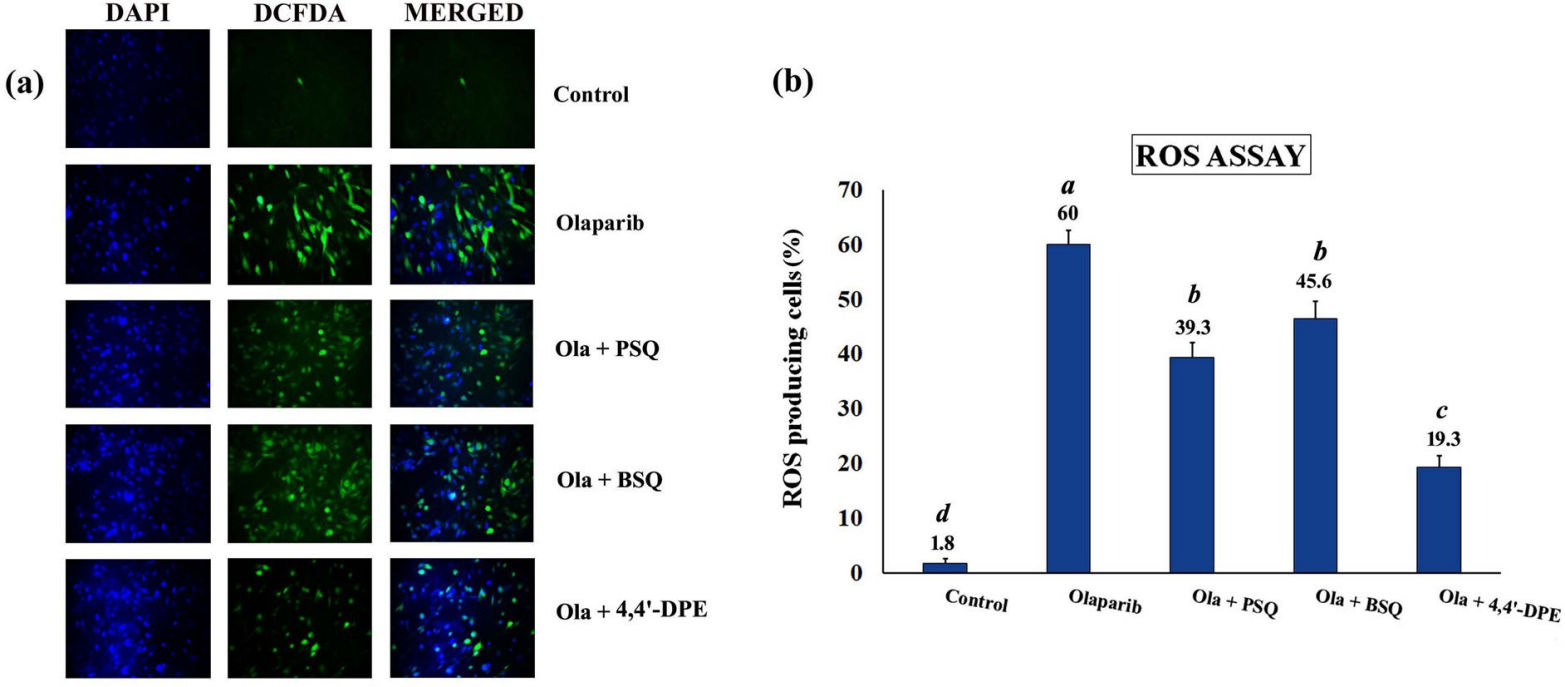
ROS assay in WI38 after exposure to 5 µg/ml olaparib for 24 h followed by application of 8 µg/ml of each test samples i.e., pure squalene standard (PSQ), bio-source squalene extracted from *Artocarpus lakoocha* L. leaves (BSQ) and squalene analog or 4,4′-diapophytofluene (4,4′-DPE) from *Cocos nucifera* L. for 48 h. (**a**) 2',7'-dichlorodihydrofluorescein diacetate (H2DCFDA) was added to WI38 cells after exposure to olaparib along with PSQ, BSQ and 4,4′-DPE. The images were captured by florescence microscopy. (**b**) Quantitative analysis of the images obtained from fluorescence microscopy using image-processing software (Image-J). Fluorescence level in the control group was arbitrarily set as 1. Data represents the mean of three replicates and error bars represent standard deviation. Letters ‘a’, ‘b’, ‘c’,‘d’ indicate significant differences (Turkey’s HSD multiple comparison at p < 0.05).

Oxidative stress was induced in WI38 cells by exposing to olaparib, followed by application of PSQ, BSQ and 4,4′-DPE treatments in separate sets to evaluate ROS scavenging activity. The percentage of ROS producing cells was increased to 62.6% in WI38 cells but when PSQ, BSQ and 4,4′-DPE were applied for 48 h, the overall percentage of ROS were significantly reduced to 39.3%, 45.6% and 19.3% respectively (Fig. 5). The treatment of 4,4′-DPE had showed apparently greater potential to scavenge ROS generated by olaparib treatment and thus may prevent senescence induction potentially compared to PSQ and BSQ.

**Figure 6:**
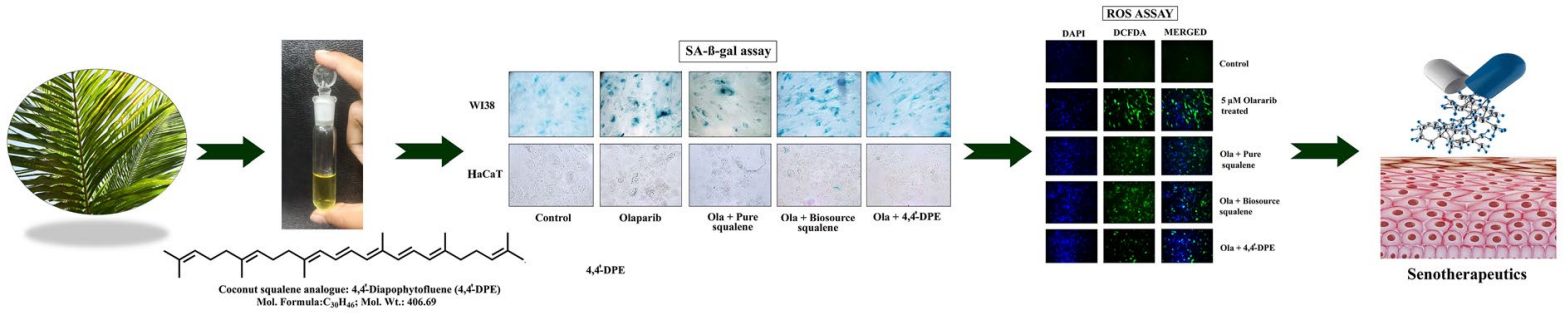
Schematic representation of overall findings of the research highlighting the activities and applications of 4,4’-diapophytofluene from leaves of *Cocos nucifera* L.

Overall findings indicated that isolated 4,4’-diapophytofluene of *Cocos nucifera* exhibited superior antisenecence activity compared with squalene (Fig. 6).

## Discussion

There has been a rising interest in the development of natural senotherapeutics, with a particular focus on testing their safety and effectiveness as anti-aging medications in recent clinical trials^25^. There are very few reported compounds such as plant polyphenols like fisetin^26^, quercetin and curcumin^27^ which can act as senotherapeutics. Squalene is also a famous natural anti-ageing compound, for its significant activities as an antioxidant, anti-inflammatory and anti-atherosclerotic agent in vivo and in vitro environment. However, its activity against senescence has not still been explored so far.

The spectral analysis (LC-HRMS, ^13^CNMR, ^1^HNMR and FTIR) confirmed the presence of 4,4’-diapophytofluene (4,4′-DPE) in pentane extract of *C. nucifera* leaves, which is a structural analog of squalene with an exception of two extra double bonds. Squalene is an intermediate metabolite in the synthesis of sterols through two major biosynthesis processes, which are mevalonic acid (MVA) pathway and methyl-erythritol-phosphate (MEP) pathway. A two-step condensation reaction between two molecules of farnesyl diphosphate in a head-to-head manner in the presence of squalene synthase (SQS) condensed into presqualene diphosphate (PSPP), which is further reduced to squalene by using nicotinamide adenine dinucleotide phosphate (NADPH) as the reducing agent^7^. This SQS gene is ubiquitous and conserved throughout the plant kingdom. However, its expression differs across tissues and conditions^28^. Parallel to this, another biosynthesis pathway is going on, i.e., carotenoid biosynthesis in plants and staphyloxanthin biosynthesis in bacteria. Farnesyl pyrophosphate converted into squalene and subsequently forms different sterols. As already discussed, PSPP present between farnesyl pyrophosphate and squalene as an intermediate, which can also be converted into dehydrosqualene instead of squalene and subsequently form staphyloxanthin. – yellowish carotenoid. This 4,4′-diapophytofluene (4,4′-DPE) is one of the intermediate of this staphyloxanthin pathway. This mentioned pathway was first reported in *Staphylococcus aureus,* but it is not reported in plants still now. Activation of crtM and crtN genes found in *Staphylococcus* sp.^29^ are mainly responsible for the formation of this 4,4′-DPE (a squalene analog). Comparing these two genes with plant genome, crtM gene showed genetical similarity with squalene synthase (SQS) and phytoene desaturase (PDS) gene ^30,31^. Though this is just a preliminary idea, a detailed study is required for the better understanding this phenomenon. In this present study, the cell viability was observed to be increased at different point of time after the senescence-induced cells were treated with PSQ and BSQ and 4,4′-DPE. However, the compound extracted from the pentane fraction of *C. nucifera* leaves (4,4′-DPE) worked more potentially to increase cell viability of the senescence induced human keratinocytes (HaCaT) up to 48 h than both bio-source^19^ and commercially available pure squalene (PSQ and BSQ). On the other side, SA-β-gal assay suggested that 4,4′-DPE exhibited better anti-senescence potentialities on both of the selected cell lines (WI38 and HaCaT) than BSQ and almost similar level of activity like PSQ. However, from ROS assay it was observed that 4,4′-diapophytofluene (4,4′-DPE) exhibited to have superior antioxidant potentialities than other two different treatments of squalene (BSQ and PSQ). It has already been found that, antioxidants are potential source of treatment to prevent senescence and thereby decelerate cellular ageing^32,33^. Several natural products have been suggested which act as antioxidants and help to prevent ageing including skin ageing. Overall, the results of this study suggested that both 4,4′-DPE and squalene were likely to have potent senescence inhibiting activity but 4,4′-DPE is more effective than squalene. This senescence inhibiting property was observed by following the mechanism of increasing the cell viability and reducing oxidative stress of the cellular environment. However, further study is needed to apply them as drugs to improve human health. Pharmacologically addressing the fundamental mechanisms of aging will have the potential to concurrently diminish the severity or delay the emergence of various age-associated co-morbidities^34^.

## Conclusion

In recent years, developing senotherapeutics from sustainable natural sources has been an emerging topic of research for rejuvenation and healthy aging. The compound named 4,4′-diapophytofluene (4,4′-DPE) was isolated and identified from the pentane fraction of *Cocos nucifera* leaf extract. Structurally, it is a squalene analog having two additional double bonds at the 8th and 12th carbon position compared with squalene. This isolated 4,4′-DPE revealed greater effectiveness than squalene in combating senescence by improving cell viability and lowering intracellular ROS levels. Therefore, it could serve as a potent senotherapeutic agent for pharmaceuticals and dermatological products. Since numerous natural products are currently employed as anti-aging agents in the cosmetic industry, it will be an additional compound with greater senotherapeutic activities. Moreover, large-scale production of this valuable compound is feasible due to its abundant, cost-effective, and sustainable natural source.

### Supplementary Information

Supplementary Figures.

## Data Availability

Data and material will be available upon request to corresponding author.
