# Supplementary material for: A squalene analog 4,4′-diapophytofluene from coconut leaves having antioxidant and anti-senescence potentialities toward human fibroblasts and keratinocytes
Source: Sci Rep. 2024 Jun 1;14:12593. doi: 10.1038/s41598-024-63547-1 (PMC11144250; doi:10.1038/s41598-024-63547-1)

**Supplementary Files**

**4, 4'- Diapophytofluene - a squalene analogue, from leaves of *Cocos nucifera* having antioxidant and anti-senescence potentialities toward human fibroblast and keratinocytes**

***^a^Madhurima Dutta^*^, ^b^Swarupa Sarkar^*^, ^b^Parimal Karmakar^©^ and ^a^Suparna Mandal Biswas^©^***

^a^Agricultural and Ecological Research Unit, Indian Statistical Institute,

203, B.T. Road, Kolkata 700108, INDIA

Tel: (+91)(033)25753225, Fax: (+91)(033)25753049

Email: [mondalsupa@gmail.com](mailto:mondalsupa@gmail.com)

^b^Department of Life Science and Biotechnology, Jadavpur University

188, Raja S.C. Mallick Rd, Kolkata 700032, INDIA

Email: pkarmakar_28@yahoo.co.in

Coconut (*Cocos nucifera)* leaves, an unutilized resource, enriched with valuable bioactive compounds. Spectral analysis of purified pentane fraction of coconut leaves revealed the presence of a squalene analog named 4,4'-diapophytofluene or in short 4,4'-DPE (C_30_H_46_). Pure squalene standard (PSQ) showed cytotoxicity after 8 µg/ml concentration whereas 4,4'-DPE exhibited no cytotoxic effects up to 16 µg/ml concentration. On senescence-induced WI38 cells, 4,4'-DPE displayed better percentage of cell viability (164.5% at 24 hrs, 159.4% at 48 hrs and 148% at 72 hrs) compared to PSQ and BSQ (bio-source squalene) with same time duration. Similar trend of result was found in HaCaT cells. SA-β-gal assay showed that number of β-galactosidase positive cells were significantly decreased in senescent cells (WI38 and HaCaT) after treated with 4,4'-DPE than PSQ, BSQ. Percentage of ROS was increased to 60% in WI38 cells after olaparib treatment. When PSQ, BSQ and 4,4'-DPE were applied separately on these oxidative-stress induced cells for 48 hrs, the overall percentage of ROS was decreased to 39.3%, 45.6% and 19.3% respectively. This 4,4'-DPE was found to be more effective in inhibiting senescence by removing ROS as compared to squalene. Therefore, this 4,4'-DPE would be new potent senotherapeutic agent for pharmaceuticals and dermatological products.

**Keywords** Squalene; 4,4'-Diapophytofluene (4,4'-DPE); Cellular senescence; Cytotoxicity, Reactive oxygen species (ROS); Senotherapeutic agent.


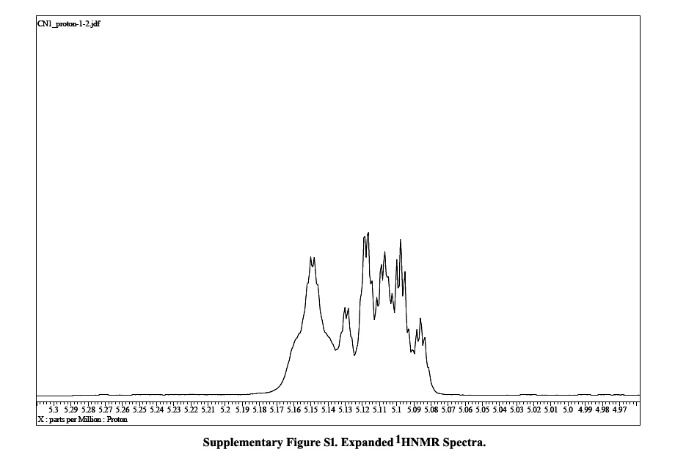


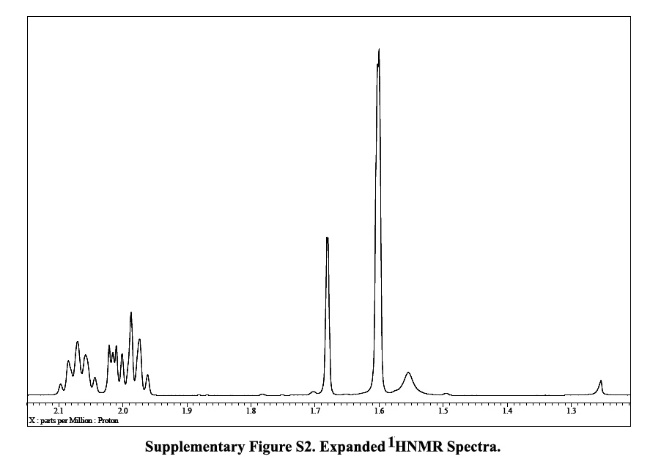


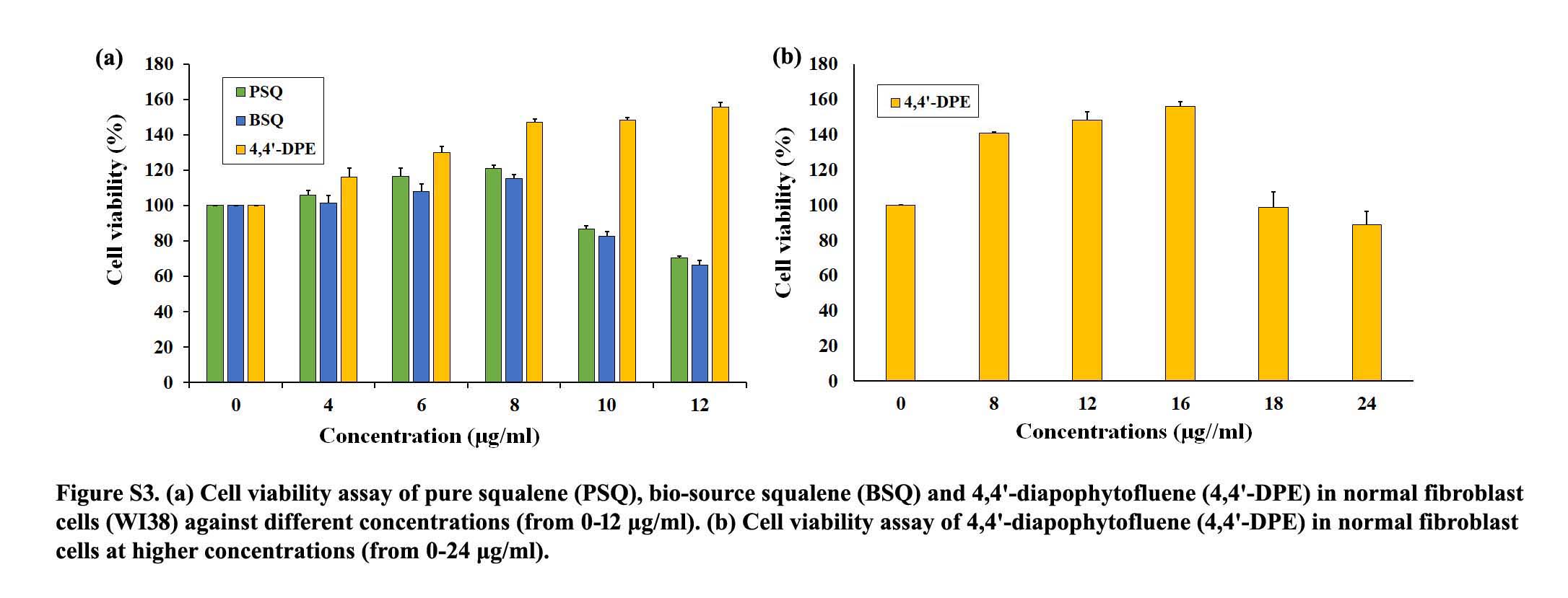

Supplement: Supplementary file 1 — Supplementary Figures. [file 41598_2024_63547_MOESM1_ESM.docx]
